# Evaluation of the structure, autoimmunity, and functions of the thyroid gland in familial Mediterranean fever patients

**DOI:** 10.20945/2359-3997000000198

**Published:** 2020-03-04

**Authors:** Müge Özsan Yilmaz, Muhammet Murat Çelik, Fatma Öztürk KelesÇ, Oguzhan Özcan

**Affiliations:** 1 Endocrinology and Metabolism Department Hatay Mustafa Kemal University Faculty of Medicine Hatay Turkey Endocrinology and Metabolism Department, Hatay Mustafa Kemal University Faculty of Medicine, Hatay, Turkey; 2 Internal Medicine Department Hatay Mustafa Kemal University Faculty of Medicine Hatay Turkey Internal Medicine Department, Hatay Mustafa Kemal University Faculty of Medicine, Hatay, Turkey; 3 Radiology Department Hatay Mustafa Kemal University Faculty of Medicine Hatay Turkey Radiology Department, Hatay Mustafa Kemal University Faculty of Medicine, Hatay, Turkey; 4 Biochemistry Department Hatay Mustafa Kemal University Faculty of Medicine Hatay Turkey Biochemistry Department, Hatay Mustafa Kemal University Faculty of Medicine, Hatay, Turkey

**Keywords:** Thyroid, familial Mediterranean fever, elastosonography, autoimmunity

## Abstract

**Objective:**

Familial Mediterranean fever (FMF) is an autosomal recessive autoinflammatory disorder that is frequently seen in the eastern Mediterranean region. The thyroid gland can be affected in FMF patients through autoimmunity or amyloidosis. Here, we aimed to evaluate the structure and functions of the thyroid gland in addition to possible autoimmunity in FMF patients.

**Subjects and methods:**

The study was conducted by the Endocrinology and Metabolism and Internal Medicine Departments. Thirty FMF patients and 30 age and gender-matched healthy controls were enrolled in the study. Free thyroxin (fT4), free triiodothyronine (fT3), thyroid-stimulating hormone (TSH), and anti-thyroid peroxidase (anti-TPO) autoantibodies were investigated. Detailed thyroid grayscale and Doppler Ultrasonography examinations and shear-wave elastosonography (SWE) were performed in the patient and control groups.

**Results:**

Anti-TPO was detected in 24% (n = 7) of the patients. On the grayscale US, mean thyroid volumes were similar between the FMF and the control groups (p > 0.05). By Doppler US, thyroid vascularity observed was detected in 10.3% (n = 3) of the patients. SWE revealed that the mean velocity value of right vs. left lobe in the patient group was 1.77 ± 0.45 m/s and 1.95 ± 0.51 m/s, respectively. Compared to the control group, the mean velocity values were significantly higher in the right (p = 0.004) and left (p = 0.01) lobes of the patient group. The mean stiffness value in the patient group was also significantly higher in the right and left lobes [10.13 ± 5.65 kPa (p = 0.005) and 12.24 ± 6.17 kPa (p = 0.02), respectively].

**Conclusion:**

Recognizing the complications of FMF early in the course of the disease is as important as the early diagnosis of the disorder. Based on this, thyroid functions and changes in its structure should be evaluated carefully for early diagnosis of a possible coexisting thyroid disorder. Arch Endocrinol Metab. 2020;64(1):66-70

## INTRODUCTION

Familial Mediterranean fever (FMF) is an autosomal recessive autoinflammatory disorder, in which patients have recurrent attacks of fever, peritonitis, synovitis, pleuritis, and pericarditis, and on rare occasions, meningitis ( 1 ). FMF is most commonly seen in eastern Mediterranean countries. The prevalence of FMF in these countries varies between 1/200 and 1/1000 ( 2 ). The incidence of FMF and the frequency of FMF-associated amyloidosis are quite high in Turkey ( 1 , 2 ). The initial diagnosis of FMF relies on clinical suspicion and is based on clinical criteria that consist of acute and reversible serosal attacks, positive family history, and detection of mutations by genetic testing ( 3 ). Autosomal recessive monogenic inheritance is relevant for the disease. The gene that is responsible for the disease is located in the short arm of chromosome 16 ( 4 ).

Based on current knowledge, there are two possible mechanisms by which the thyroid gland might be affected in FMF. First, systemic amyloidosis might involve the thyroid gland in FMF patients. Due to this rarely seen occasion of diffuse amyloid deposition, the thyroid might rapidly develop into a diffusely enlarged gland, which leads to dyspnea, dysphagia, and hoarseness, which might mimic malignancy ( 5 ). In the second form, the thyroid gland in FMF is affected asymptomatically, and other organs are affected by systemic amyloidosis ( 6 ).

Thyroid autoimmunity is characterized by the infiltration of the thyroid by inflammatory cells and elevation of the serum thyroid antibodies, such as anti-thyroid peroxidase and anti-thyroglobulin antibody. As a consequence of these disturbances, some structural modifications might develop in the thyroid gland ( 7 ). Autoimmune thyroiditis results in either thyroid hyperfunction (as in Grave’s disease) or hypofunction by the destruction of thyroid structure (as in Hashimoto’s Disease) ( 8 ). Autoimmune thyroid diseases are commonly seen in some rheumatic diseases ( 9 ). However, FMF is an autoinflammatory rheumatic disease but can overlap with autoimmune disorders (e.g., autoimmune thyroid disease), which share certain inflammatory markers ( 10 ). Only a few case reports have addressed the co-occurrence of autoimmune thyroid disease and FMF; however, it has been suggested that FMF aggravates the autoimmune response. Therefore, in FMF, autoimmune thyroid disease might be seen more frequently than in the healthy population.

In this study, we investigate whether there are structural changes in the thyroid of FMF patients using shear wave elastosonography and compare the status and autoimmunity of the thyroid gland with the healthy controls in our geographical region, which is a prominent location for FMF.

## SUBJECTS AND METHODS

The study was conducted by the Endocrinology and Metabolism and Internal Medicine Departments of Hatay Mustafa Kemal University School of Medicine. Thirty FMF (diagnosed using the Tel Hashomer’s criteria) patients and 30 age- and gender-matched healthy controls were enrolled in the study. Patients with established thyroid disorder were excluded from the study. Written informed consent was obtained from all patients, and the study was reviewed and approved by the Medical Ethics Committee of Hatay Mustafa Kemal University. Demographical data (e.g., age, gender, and smoking status) and FMF-related parameters (e.g., the onset age and the duration of the attacks and medications used for treatment) were recorded. Physical examination, especially for the presence of a palpable goiter mass, was performed on every patient.

After at least 12 h of overnight fasting, blood samples were collected from study and control subjects. All samples were centrifuged at 1,500 × g for 10 min. After separation, serum samples were aliquoted and stored at -80°C until the time of assay. Serum-free thyroxin (fT4), free triiodothyronine (fT3), and thyroid-stimulating hormone (TSH) levels were assayed by an electrochemiluminescence method using commercially available kits (Siemens Advia Centaur XP, USA). The normal reference range for TSH is 0.57-5.60 mIU/L, for fT3 is 2.60-4.80 pg/mL, and for fT4 is 0.80-2.30 ng/dL. Serum anti-thyroid peroxidase (anti-TPO) autoantibodies were studied by chemiluminescence method (Immulite 2000 XPi, USA) and the results were recorded. The reference value for anti-TPO (IU/mL) was 0-35 IU/mL. Serum anti-TPO levels higher than 35 IU/mL were considered positive.

**Figure 1:**
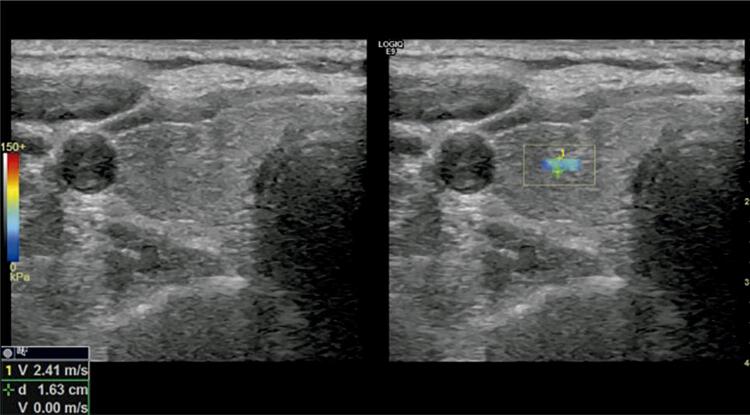
In the patients group, the thyroid gland shows in the right lobe the axial plan of the velocity value.

**Figure 2:**
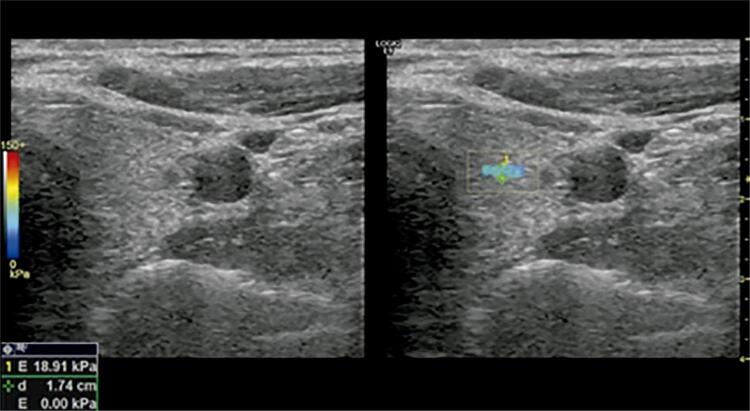
In the patients group, the thyroid gland shows in the left lobe the axial plan of the stiffness value.

Detailed thyroid grayscale and Doppler US examinations and elastosonography were performed by the same radiologist in Hatay Mustafa Kemal University School of Medicine Radiology Department. Radiologic studies were evaluated by Logiq E9 shear-wave elastosonography (Logic E9 XDClear, GE Healthcare, Milwaukee, WI, USA) by 9 MHz linear transducers. The patients were examined in the supine position with their neck in mild hyperextension by application of gel at room temperature. Multiple US parameters were recorded: the thyroid gland volume (craniocaudal × anterior-posterior × transverse dimensions × 0.52), the structure and echogenicity of the parenchyma, presence of micronodulation, and contour irregularity. During the Doppler US examination, thyroid vascularization was also evaluated. Shear-wave elastosonography (SWE) measurements were obtained under normal breathing conditions using a linear probe without compression application. Full attention was given to achieving stillness of the hand of the operator who was taking the images. Three different measurements were obtained from each lobe at the axial plane, which was distant from the vascular structures and with a standard 2-mm region of interest (ROI) while the probe was perpendicular to the parenchyma. The measurements in both thyroid lobes were made from the center of the lobe with the possible furthest distance from the main carotid artery. Stiffness and velocity values were obtained in every ROI in kilopascal (kPa) and m/s, respectively, and the mean value was recorded for each thyroid lobe ( Figures 1 and 2 ).

### Statistical analysis

After all data were recorded, calculations were made using the SPSS 21 Statistics Program. Descriptive statistics were calculated as mean, standard error, number, and frequency. The comparisons of demographic data between groups were analyzed by the Chi-square test. The Kolmogorov-Smirnov and Shapiro-Wilk tests were used to assess the normal distribution of numeric data. Correlations between numeric values were analyzed using Spearman or Pearson correlation analyses. T-test and Mann-Whitney tests were used as multiple comparison methods. A p-value lower than 0.05 was accepted as statistically significant.

## RESULTS

Thirty (16 male, 14 female) FMF patients and 30 healthy volunteers (16 male, 14 female) comprising the control group were enrolled in the study. Based on physical examinations, goiter was not detected in the patient group. The distribution of age across the FMF and the control groups was similar (28.6 ± 1.1 and 30.9 ± 11.8, respectively) (p > 0.05) ( Table 1 ). The median time of disease duration was 13.5 years (minimum, 3 years; maximum, 37 years) in the patient group, and all the patients were on remission for FMF. Anti-TPO antibody levels were found to be slightly elevated [mean 12.6 ± 5.8 (0-35) IU/mL] over the normal range in 24% (n = 7) of the patients ( Table 1 ). On grayscale US, the mean thyroid gland volume was 6.37 ± 4.72 mL (minimum, 1.51 mL; maximum, 26.52 mL) in the right lobe and 5.31 ± 3.02 mL (minimum, 1.4 mL; maximum, 16.07) in left lobe in FMF patients. Mean thyroid volumes, which were 5.27 ± 1.92 in the right and 4.37 ± 1.57 in the left lobe, were similar between FMF and control groups (p = 0.35, p = 0.27 respectively). The parenchymal structure was heterogeneous in 41.4% (n = 12) of the patient group. Micronodulation with non-malign features was observed in 33.3% (n = 9) of the patients. The US findings of thyroid autoimmunity, which consisted of contour irregularity, pseudonodulation, and intraparenchymal hyperechoic septa, were not present in the patient group. Thyroid vascularity in Doppler US was elevated in 10.3% (n = 3) of the patients ( Table 2 ). Mean velocity values of the right and the left lobes in the patient group were 1.77 ± 0.45 m/s and 1.95 ± 0.51 m/s, respectively. Mean velocity values of both lobes observed via shear-wave elastosonography in the patient group were significantly higher than those of the control group (p = 0.004 and p = 0.01, respectively). The mean stiffness values were 10.13 ± 5.65 kPa in the right lobe, and 12.24 ± 6.17 kPa in the left lobe, which were also significantly higher than those of the patient group (p = 0.005 and 0.02, respectively) ( Table 2 ).

**Table 1:** Demographic features and biochemical findings of the patient and control groups

	Control group (n = 30)	Patient group (n = 30)	p
Age (year ± SD)	28.6 ± 1.1	30.9 ± 11.8	0.83
Gender (male/female)	16/14	16/14	1
TSH (mIU/L) ± SD	1.67 ± 0.54 (0.57-5.60)	1.84 ± 1.34 (0.57-5.60)	0.53
Free T4 (ng/dL) ± SD	1.12 ± 0.21 (0.80-2.30)	1.14 ± 0.14 (0.80-2.30)	0.61
Free T3 (pg/mL) ± SD	3.23 ± 0.39 (2.60-4.80)	3.23 ± 0.47 (2.60-4.80)	0.95
Anti-TPO (IU/mL) (min-max)	0-15 (0-35)	0-34 (0-35)	0.22

SD: standard deviation; TSH: thyroid-stimulating hormone; anti-TPO: anti-thyroid peroxidase.

**Table 2:** Thyroid US and SWE findings of patient and control group

Thyroid US (Gray scale)	Control group (n = 30)		Patient group (n = 30)		p
Parenchymal heterogeneity	0/30		12/30 (41.4%)		< 0.01
Micronodulation	0/30		9/30 (33.3%)		0.001
Increased vascularity	0/30		3/30 (10.3%)		0.07
Elastosonography (Shear-wave)	Right	Left	Right	Left	p*
Volume (mL)	5,27 ± 1.92	4.37 ± 1.57	6.37 ± 4.72	5.32 ± 3.02	0.35/0.27
Velocity (m/s)	1.43 ± 0.27	1.65 ± 0.29	1.77 ± 0.45	1.95 ± 0.510	0.004/0.016
Stiffness (kPa)	6.53 ± 2.50	8.67 ± 2.83	10.13 ± 5.65	12.24 ± 6.17	0.005/0.021

US: Ultrasonography; SWE: Shear-wave elastosonography; p* between control and patient groups’ left and right lobes.

AntiTPO antibody levels were inversely correlated with the volume of the right thyroid lobe (p = 0.007, r = -0.48). There was no correlation between SWE findings and the duration of the disease, TSH, fT4, fT3, and AntiTPO. Significant correlations were found between age and left thyroid velocity and stiffness (p = 0.003, r = 0.57 and p = 0.004, r = 0.56, respectively).

## DISCUSSION

FMF is an inherited multi-system disease with recurrent painful attacks that affect the chest, the abdomen, and the joints, and is often accompanied by fever ( 11 ). It is inherited in an autosomal recessive mode. The disorder is mostly seen in Sephardic Jewish, Armenian, Levantine Arabic, and Turkish people ( 12 ). The FMF gene, which is designated as MEFV (Mediterranean fever), has been mapped to the short arm of chromosome 16, where a protein called pyrin (which is involved in inflammation) is also coded. Mutations in the pyrin gene form the genetic basis of FMF pathogenesis. MEFV mutations cause increased interleukin (IL)-1 secretion, which maintains the inflammatory processes in FMF ( 4 , 12 ).

FMF has been reported to coexist with autoimmune disorders, such as polyarteritis nodosa, overlap syndrome, and multiple sclerosis ( 13 - 16 ). Autoinflammatory disorders share many features with autoimmune diseases. For example, FMF and HT have common inflammatory markers, such as interleukins (ILs) and tumor necrosis factor (TNF)-α ( 17 ). From this point of view, Dikbas and cols. studied the prevalence of thyroid autoimmunity in FMF patients and reported that thyroid autoimmunity was more frequent in their study group ( 18 ). However, in our study, we found similar antiTPO levels when comparing the patient and control groups. Nevertheless, the higher incidence of parenchymal heterogeneity (41.4%) as estimated using grayscale US detected in the patient group might be a reflection of autoimmune thyroiditis.

In other respects, thyroid heterogeneity on echotexture might be an early indicator of amyloidosis. Amyloidosis, in which there is a deposition of insoluble material in various organs, is a complication of FMF. The thyroid gland might be asymptomatically involved by the amyloid substance in nearly 30%-80% of the patients with primary or secondary amyloidosis ( 19 ). Thyroid enlargement with diffuse amyloid deposits is a rare manifestation of primary or secondary amyloidosis ( 20 ). Although amyloid goiter is rare, the frequency of focal amyloid deposits ranges between 30% and 80% ( 21 ). In our patients, increased heterogeneity rates were associated with amyloidosis; however, we do not have data for thyroid biopsies. This was the limitation of our study.

In recent years, a new elastography technique, which is called SWE and provides an estimation of tissue stiffness, has been introduced into clinical practice for thyroid diseases ( 22 ). SWE has been frequently used in nodular thyroid disease; however, its use in diffuse thyroid diseases has been reported in a small number of studies ( 22 , 23 ). Our study, to the best of our knowledge, is the first in which SWE has been used for thyroid evaluation in FMF patients. We obtained significant results, such as mean velocity and stiffness values, that were both higher in the patient group compared to the control group. SWE changes might be associated with amyloidosis; nevertheless, the presence of amyloidosis has to be confirmed by thyroid aspiration biopsy ( 19 ).

In FMF, the thyroid can be affected by two distinct processes. First, the thyroid might be affected by amyloid in diffuse or focal patterns, as discussed in the previous paragraph ( 20 , 21 ). The second process affecting the thyroid is autoinflammation. However, we did not observe any signs and symptoms of amyloidosis in our patients; additionally, markers of thyroid autoinflammation were not significantly elevated in the patient group. There was no correlation between the duration of the disease and the SWE findings. A single positive relationship was found between the age and the left thyroid velocity and stiffness. Macro and micronodulation, fibrotic changes, increased lymphocyte quantity, and decreased colloid amount can be relevant to thyroid aging ( 24 ). These changes might be reflected on SWE as increased velocity and stiffness.

Conclusively, FMF is a common disorder in Turkey and is seen in high frequencies, especially in the Mediterranean region. Early recognition of FMF complications is as important as the early diagnosis of the disease. In these circumstances, structural changes and functions of the thyroid should be evaluated carefully in FMF patients. Thyroid SWE is a novel noninvasive technique for the evaluation of thyroid. Thyroid biopsy can be performed in patients who have predictive values on SWE. We propose that amyloidosis might be diagnosed earlier by using this approach.
